# A QUALITATIVE STUDY ON THE EXPERIENCE OF SMART CARE TECHNOLOGY UTILIZATION AMONG OLDER ADULTS IN KOREA

**DOI:** 10.1093/geroni/igae098.2985

**Published:** 2024-12-31

**Authors:** Sungwan Kang, Boram Hwang, Haklyoung Kim, Seongsu Choi, Kyoungmi Mun, Yeong Ju Lee

**Affiliations:** Pusan National University, Busan, Republic of Korea; Pusan National University, Busan, Republic of Korea; Pusan National University, Busan, Republic of Korea; Pusan National University, Busan, Republic of Korea; Pusan National University, Busan, Republic of Korea; Pusan National University, Busan, Republic of Korea

## Abstract

To address the increasing desire among older adults who live alone to remain in the community, the Korean government and Korean companies have implemented pilot projects aimed at expanding smart technology integrated care services. Evaluations of these efforts have primarily focused on cost savings and the number of emergency rescue cases through the replacement of care personnel. However, there is a lack of research on users’ experiences with various smart technologies. In particular, research on the experience of using services based on artificial intelligence (AI) and Internet of Things (IoT) among low-income, low-educated older adults remains limited. The purpose of this study is to explore the experiences of low-income, low-educated older adults on using AI-based emotional support services and IoT-based abnormal symptom monitoring services. We conducted focus group interviews with 13 participants who used the smart care services for three months in Korea. Using thematic analysis, three main themes emerged: (1) emotions and values, which represent ambivalence toward artificial intelligence (gratitude, connection, indifference, disconnection). (2) social support, which describes the alleviation of negative emotions; government workers including social workers provided detailed explanations about the services, including purposes, usage procedure, and addressing concerns related to the system. (3) new experiences, which discuss the usefulness and value that were newly discovered over the period of use; study participants emphasized that they could lessen their burden on family members. These findings serve as foundational insights for the development and dissemination of smart care services tailored to the needs of low-income, low-educated older adults.

